# Multistage Static and Dynamic Optimization Framework for Composite Laminates in Lightweight Urban Rail Vehicle Car Bodies

**DOI:** 10.3390/ma19030531

**Published:** 2026-01-29

**Authors:** Alessio Cascino, Francesco Distaso, Enrico Meli, Andrea Rindi

**Affiliations:** Department of Industrial Engineering, University of Florence, 50139 Florence, Italy

**Keywords:** railway vehicle design, railway dynamics, finite element analysis, multilayer composite panels, lightweight design, structural optimization, composite materials

## Abstract

This paper presents a robust multistage optimization framework for the integration of composite laminates into the car body shell of a low-floor light rail vehicle (LRV). While structural design in low-floor vehicles is typically complex, this methodology successfully balances both static and dynamic requirements through a sequential optimization process. Developed in strict accordance with reference European standards, the methodology addresses the structural challenges inherent in low-floor architectures, where complex load paths and redistributed equipment masses require targeted reinforcement. The proposed approach sequentially addresses dynamic and static requirements through a structural optimization process. Two distinct 10-ply laminate configurations, one symmetric and one asymmetric, were investigated. The results demonstrate that the multistage optimization successfully converged to a highly mass-efficient solution, achieving a 66% reduction in laminate thickness compared to the baseline design. This significant result was accomplished while maintaining full regulatory compliance; the failure index increased by approximately 22.5% and 23.3% for the two composite laminate configurations, respectively, effectively maximizing material utilization. A key finding of this study is the preservation of structural dynamic integrity; the fundamental natural frequency was maintained at approximately 16 Hz, with a high correlation across the first ten vibration modes, confirming that the global dynamic behaviour remains unaffected. These observations provide critical insights into the synergy between hybridization and structural constraints, suggesting a systematic pathway for designers to achieve an optimal trade-off between manufacturing costs, weight reduction, and performance in advanced urban transit platforms.

## 1. Introduction

In the modern railway industry, the pursuit of lightweight design has become a primary driver for enhancing energy efficiency and reducing life-cycle costs, particularly for urban transit systems. The integration of advanced composite materials into traditional metallic car body structures represents a strategic frontier for achieving significant mass reduction without compromising the rigorous safety and structural requirements dictated by international standards. Structural optimization has become an indispensable tool across diverse engineering disciplines, ranging from aerospace to automotive and civil infrastructure. By employing various methodologies, such as topology, shape, and sizing optimization, engineers can systematically identify the most efficient material distribution, ensuring that performance targets are met with minimal resource consumption.

While sectors like aeronautics have long integrated these processes to push the boundaries of high-performance design [1,2,3], there is a growing necessity for a similar technological evolution within the railway industry. The recent literature highlights a significant shift toward the integration of advanced materials and automated structural refinement in railway applications. Several investigations have focused on the implementation of composite and sandwich architectures for car body shells, conducting rigorous assessments of stiffness, strength, and buckling stability to enhance load-bearing efficiency. For instance, the use of finite element analysis (FEA) combined with a multilevel optimization approach has demonstrated the potential for mass savings in the region of 30% [4,5], while experimental studies on curved glass fibre-reinforced plastic (GFRP) components have validated their durability and impact performance in operational environments [6]. Beyond material substitution, modern research increasingly leverages diverse optimization paradigms. In the context of light rail transit, dynamic sizing optimization has proven essential for aligning structural mass reduction with specific modal requirements [7]. Furthermore, innovative designs have explored the integration of honeycomb core panels to push the boundaries of car body weight efficiency even further [8,9,10]. These developments underscore the growing trend toward hybrid, optimized structures that reconcile mechanical robustness with the stringent weight constraints of modern transit systems. The adoption of composite laminates has become diffuse across the aerospace, automotive, and maritime sectors, driven by their exceptional specific strength, inherent corrosion immunity, and the unique possibility of structural tailoring to meet precise operational demands. In the aeronautical field, specialized solutions such as fibre metal laminates (FMLs), including GLARE and ARALL, are extensively integrated into fuselage sections and primary structural panels. These materials are specifically selected for their superior fatigue life and high energy absorption during impact events [11,12,13]. Similarly, the automotive industry leverages these advanced materials for both primary load-bearing structures and secondary components, such as exterior bodywork, lighting housings, and internal cabin elements. This integration is pivotal for the development of lightweight, fuel-efficient vehicles that do not compromise on crashworthiness or long-term durability [14,15,16,17]. Furthermore, the marine sector exploits the environmental resilience of composite systems, utilizing them for hulls, decking, and superstructures to withstand the aggressive corrosive nature of saltwater environments [18,19,20]. With reference to the integration of carbon-fibre-reinforced polymers (CFRPs), they offer a superior alternative for aggressive weight-saving targets. Recent research has shifted focus toward evaluating how these composite solutions compare to traditional aluminium frames, particularly concerning their long-term structural integrity, fatigue life, and the total cost of ownership within the railway sector [21,22,23,24,25,26]. However, aluminium alloys still represent the primary choice for car body structures due to their reliable mechanical properties and established fabrication techniques [27]. The implementation of composite laminates is not without significant hurdles; they exhibit intricate failure mechanisms, such as interlaminar debonding and matrix degradation, alongside substantial manufacturing expenditures and the requirement for specialized maintenance protocols. To address these limitations, current scientific efforts are centred on optimizing production scalability, exploring sustainable alternatives like hybrid and bio-based reinforcements, and advancing structural health monitoring (SHM) through sophisticated computational algorithms [28,29,30,31]. The scope of lightweight engineering reaches beyond the car body shell to include other safety-critical assemblies, such as bogie frames and bolster beams. In these high-stress components, the synergy between advanced material selection and structural optimization has proven effective in achieving substantial mass reduction while maintaining full compliance with the EN 13749 regulatory framework [32,33,34,35,36,37]. The pursuit of energy-efficient rolling stock has led to a widespread adoption of finite element analysis (FEA) to validate structural performance under real-world conditions. The recent literature underscores the potential of this approach: characterizing tailored composite architectures has yielded mass savings of 28% over aluminium alloys while preserving fatigue and impact resistance [38]. Furthermore, the implementation of pultruded GFRP panels has demonstrated even greater efficiency, achieving a 35.5% weight reduction compared to conventional steel assemblies [39]. In this perspective, the continuous monitoring of vehicle behaviour in service also plays a crucial role, providing real-world data to enable increasingly accurate design and optimization strategies for both passenger and freight transport systems made with traditional and innovative materials [40,41,42,43,44,45]. These benchmarks confirm that advanced numerical modelling is essential for transitioning from traditional metallic designs to high-performance composite structures. Finally, while advanced composite laminates represent a critical technological frontier for the railway industry, offering unprecedented opportunities for mass reduction, their full potential can only be realized through systematic optimization procedures integrated into the component development phase. To ensure both speed and efficiency in the design process, these methodologies must strictly align with established European regulatory standards. In this context, the research presented in this paper aims to bridge this specific gap by proposing a multistage optimization framework capable of simultaneously balancing dynamic behaviour and static strength requirements for low-floor architectures. These systems are characterized by unique structural complexities, such as discontinuous load paths and the concentration of heavy equipment on the roof, which demand highly specialized optimization strategies. By automating the convergence toward high-performance configurations while ensuring full regulatory compliance, this work provides a reliable methodology for the development of the next generation of lightweight and sustainable urban transit platforms.

## 2. Methodology

In this section, the multistage optimization framework proposed for composite laminates in railway car body applications is detailed. The methodology, developed in compliance with relevant European standards (e.g., EN 12663-1 [46], EN 15663 [47], EN 45545 [48]), aims to balance dynamic performance and structural integrity with mass reduction. To evaluate the performance of the proposed optimization framework, two distinct composite laminate configurations were investigated. Both configurations feature a 10-ply architecture but are differentiated by their structural arrangement:

- Configuration 1 (Symmetric) utilizes a symmetric stacking sequence relative to the mid-plane to eliminate extension–bending coupling, ensuring structural stability under pure in-plane loads.
- Configuration 2 (Asymmetric) employs a non-symmetric layup to explore the influence of coupling effects on the overall car body response and to test the flexibility of the multistage optimization algorithm in handling more complex constitutive behaviours.

The overall workflow can be summarized as follows:

1. The initial phase of the methodology involves the validation of a comprehensive finite element (FE) model of the railway vehicle. In accordance with the EN 12663-1 standard, load cases are applied to the full vehicle assembly rather than an isolated car body component to accurately simulate real-world operational stresses. In industrial railway engineering, the development of a new platform typically leverages inherited data from previous successful projects. Consequently, the preliminary design phase often relies on the expertise of the design team, frequently resulting in an over-dimensioned baseline model, especially for the metallic part of the car body. The primary objective of this stage is to establish a feasible baseline configuration. Ensuring that the initial structure fully complies with the safety margins dictated by European regulations is a prerequisite for the subsequent optimization. Applying an optimization algorithm to a baseline that fails to support its primary design loads would be computationally inefficient and physically inconsistent; therefore, a strong but heavy starting point is essential for the stability of the multistage process.
2. Second, starting from the FE model of the vehicle, each car body is isolated with the objective to study its modal behaviour through a modal analysis in free-free conditions. The parameter of interest is the first natural frequency of the structure. Mainly, the lowest natural mode is the vertical bending mode, but the class of the modes could depend on the properties of the system analysed: twist and compression deformation modes are also important in car body design. To avoid resonances, a common practical design rule is to keep the first natural frequency of the car body as high as possible. The first vertical bending mode, discussed and observed during the present activity, provides the main contribution to the vertical vibration in the middle part of the car body, except for the rigid vibration of the car body (below 2 Hz). Rarely, these frequencies may be below 10 Hz. Maintaining a higher frequency threshold is critical for both passenger comfort and structural integrity. From a comfort perspective, the human body exhibits peak sensitivity to vibrations within the 4–10 Hz range; therefore, decoupling the car body’s natural modes from this range is vital to prevent resonance-induced discomfort. Additionally, from a vehicle dynamics standpoint, frequencies below 10 Hz may coincide with the bogies’ hunting motion or lower-order track excitations. Such overlap could lead to detrimental aeroelastic or dynamic coupling, potentially compromising the structural stability of the car body shell. which is close to the vibration frequencies to which the human body is sensitive. Passenger comfort is always a priority. This step allows the designer to know the modal behaviour of the structure, in terms of frequency and mode shapes, which is useful when defining the constraint condition for the optimization step. If the first frequency of vibration is too low, it means that the car body needs to be redesigned with the aim of increasing this fundamental parameter.
3. The third stage of the methodology involves a multistage size optimization of the composite laminates, characterized by a hierarchical approach that sequentially addresses dynamic and static requirements. In the first phase, a dynamic sizing optimization is performed to identify the minimum allowable thickness for each ply based on the free-free modal response of the structure. The primary objective is the minimization of total mass, subject to a constraint on the fundamental natural frequency identified in the previous characterization step. The laminate thickness is allowed to vary within a predefined design range, and the resulting optimized values establish a new lower-bound threshold for the subsequent phase. This ensures that the dynamic integrity of the car body is preserved throughout the process. In the second phase, the optimization is driven by the most critical static condition for the floor and seating regions, the maximum vertical load case, as specified by the reference European standard. Using the thickness results from the dynamic stage as a baseline, the optimizer determines the final minimum ply and laminate thicknesses required to simultaneously satisfy both vibrational and strength criteria. This dual-stage strategy ensures that the final configuration is compliant with all regulatory safety margins while achieving a high degree of mass efficiency.
4. Upon the successful completion of single car body optimization, a lightweight structural configuration is achieved that adheres to the target fundamental frequency. This optimized geometry must then be integrated back into the global assembly; specifically, the original finite element model of the full vehicle is updated with the refined thickness distributions derived from the optimization of the individual components. This final iteration of the complete vehicle model undergoes a comprehensive validation phase to ensure that its mechanical performance remains in full compliance with the EN 12663-1 standard.

By applying this methodology to two distinct laminate configurations, one symmetric and one asymmetric, the robustness and versatility of the proposed framework are further validated. This comparative approach addresses a significant gap in the current literature and industrial practice regarding the design of composite laminates for railway applications, providing a systematic procedure for developing advanced materials that strictly align with established European regulatory frameworks.

## 3. Light Rail Vehicle Description

**Figure 1:**
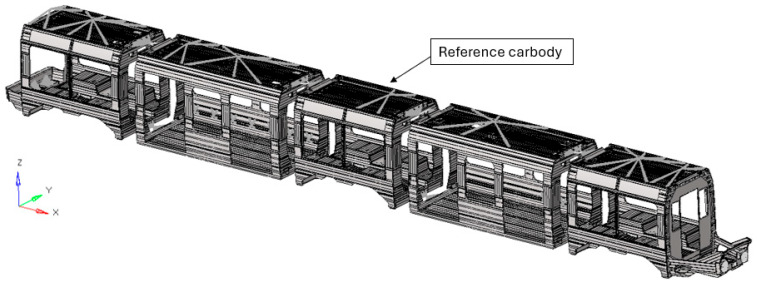
Urban rail vehicle (five car bodies configuration).

The study is based on a low-floor light rail vehicle representative of current urban tramway platforms. The vehicle architecture adopts a modular configuration composed of multiple car body units, typically five or seven, depending on the operational layout. Low-floor designs, while essential for accessibility and passenger flow, impose non-negligible structural penalties when compared to conventional high-floor solutions. The reduction in the available vertical space fundamentally alters the structural hierarchy of the vehicle, requiring alternative load paths and a redistribution of stresses within the underframe and surrounding components. Consequently, the primary structure must be locally reinforced in zones where geometric discontinuities and functional openings coexist, such as passenger access areas and articulation connections between adjacent car bodies. In addition, the absence of a raised floor eliminates the possibility of accommodating key onboard systems within the underframe. Traction and braking equipment must therefore be repositioned, most commonly on the vehicle roof or within the bogie assemblies. This design philosophy enables compliance with accessibility requirements while maintaining adequate structural stiffness, strength, and passenger capacity. In the reference configuration adopted by the manufacturer, shown in Figure 1, aluminium alloys were selected for the car body shells, composite materials were employed for the driver cab structures, and high-strength steel was used for the bogie frames.

**Figure 2:**
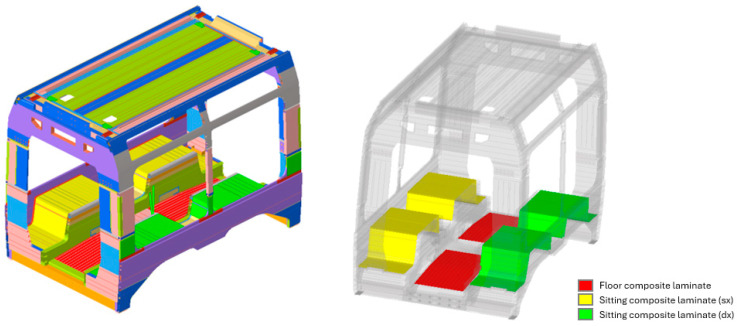
Car body FE model on the left, composite laminate positioning on the right.

Within this structural context, the introduction and structural optimization of composite materials was investigated as a means of developing a hybrid vehicle architecture. Multilayer composite laminates were selectively applied to specific components, as schematically shown in Figure 2. Several structural elements were initially considered for this purpose, including external panels, roof sections, and internal load-bearing components. The present work concentrates on the underframe and seating support structures, where the original metallic components were substituted with composite laminate solutions. This selection allowed a focused analysis of structural response and mass efficiency. Both components are subject to substantial service loads and play a relevant role in the global stiffness of the vehicle. These loading conditions are particularly well suited to the mechanical characteristics of laminated composites, which offer high specific stiffness and strength. Consequently, the adopted approach provides a meaningful opportunity to assess the potential of composite materials for structural weight reduction while preserving, or potentially enhancing, the overall mechanical performance of the vehicle.

### 3.1. Structural Materials and Composite Laminate Configurations

#### 3.1.1. Aluminium Alloy

The metallic reference solution adopted in this investigation is based on the aluminium alloy EN AW 6106 T6, selected for its widespread use in railway vehicle structures and its compliance with current European design regulations. The material properties considered in the analyses are consistent with the specifications provided by EN 1999-1-1:2014 [49]. EN AW 6106 belongs to the Al–Mg–Si alloy family, in which the combined presence of magnesium and silicon promotes precipitation hardening through the formation of Mg_2_Si phases. This metallurgical mechanism is responsible for the strength level typically associated with the 6xxx series alloys. From a structural design perspective, EN AW 6106 T6 offers a balanced combination of characteristics that are particularly attractive for transportation applications. The T6 condition, obtained via solution heat treatment followed by artificial ageing, enhances yield and ultimate strength while maintaining adequate ductility and resistance to fatigue damage. In addition, the alloy exhibits good corrosion resistance and weldability, which are essential attributes for large, welded assemblies such as railway car bodies. These features explain its extensive adoption as a baseline material in lightweight rail vehicle construction.

#### 3.1.2. Composite Laminate Configurations

In the pursuit of weight-efficient structural solutions for transportation applications, laminated composite materials have emerged as a viable alternative to traditional metallic designs. Their adoption is driven not only by favourable mechanical properties, such as high stiffness and strength at low mass, but also by their durability in aggressive environmental conditions. A defining characteristic of composite laminates is the possibility to actively design the load-carrying behaviour of a component through the selection and arrangement of individual layers. By varying material systems, fibre orientations, and layer thicknesses, the structural response can be tuned to the dominant service loads, enabling designs that simultaneously address performance requirements, weight targets, and manufacturing constraints. In this research work, the development and optimization of a tramway car body structure are addressed with specific emphasis on the definition and assessment of composite laminate configurations. Rather than focusing solely on material substitution, the study investigates how different laminate architectures can be systematically designed and integrated within a full-scale vehicle structure to meet structural, weight and regulatory requirements. The novelty of the work lies in the combined evaluation of laminate configuration, structural performance and mass efficiency at vehicle level, using a high-fidelity finite element model compliant with current railway standards. By exploring hybrid laminate solutions applied to load interior components, the proposed approach provides a structured framework for exploiting the design flexibility of composite materials in the tramway applications, offering practical insights into lightweight structural concepts that balance mechanical performance, manufacturability, and cost considerations. Two hybrid laminate configurations combining carbon-fibre-reinforced polymer (CFRP) and glass-fibre-reinforced polymer (GFRP) were developed and investigated for application in railway vehicle flooring and seating structures. The first configuration, referred to as the baseline laminate, was designed to maximize bending stiffness and overall structural stiffness, which are critical requirements for regions subjected to concentrated loads, such as passenger seat supports and equipment mounting areas. The total laminate thickness for this configuration is 3.3 mm. By positioning unidirectional CFRP plies on the outer surfaces and GFRP plies within the internal layers, including ±45° orientations, the laminate achieves a synergistic combination of high longitudinal stiffness, enhanced shear resistance, and improved energy absorption. The symmetric and balanced stacking sequence ensures a uniform structural response under flexural loading, limits residual stresses induced during curing, and reduces the likelihood of warping or delamination under service conditions. The second configuration, referred to as the cost-reduced laminate, features a reduced overall carbon fibre content and a total laminate thickness of 3.0 mm, while maintaining comparable shear toughness and energy absorption performance through an increased proportion of GFRP plies in the core region. This solution represents a balanced compromise between material cost, structural performance, and manufacturability, making it suitable for non-critical areas where stiffness requirements are moderate, while impact resistance and durability remain essential. Both laminate configurations provide effective solutions for railway floor and seat applications. The CFRP outer layers contribute significantly to stiffness and load-carrying capability, which are crucial for long-term service performance and fatigue resistance, whereas the GFRP core helps to limit the initiation and propagation of localized damage caused by impacts or repeated pedestrian traffic. In addition, the ±45° plies within the GFRP core enhance the in-plane shear strength, a particularly relevant feature under the dynamic loading conditions typical of railway operations. Overall, the proposed laminate designs combine lightweight construction, high structural efficiency, and resistance to localized impacts, thereby satisfying the primary functional requirements of modern railway interior structures while offering flexibility in material selection to address cost–performance trade-offs. The mechanical properties and the failure parameters values of each ply and the corresponding materials are summarized in Table 1, Table 2 and Table 3.

A comparative assessment of the two laminate configurations highlights the different design priorities adopted. Configuration 1 prioritizes bending stiffness and load-carrying capacity; as shown in Table 4, its higher carbon concentration ensures superior specific stiffness, making it particularly suitable for structurally critical regions. Configuration 2, on the other hand, emphasizes cost reduction through a strategic hybridization approach. In this case, the carbon fibre content is significantly reduced by more than 75% (dropping from 36.36% to 8.33%), resulting in a ‘glass-intensive’ solution. This transition leverages the higher strain-to-failure ratio of glass fibres to preserve shear toughness and impact energy absorption while substantially decreasing raw material costs. In both cases, laminate symmetry plays a key role in ensuring predictable mechanical behaviour and reducing manufacturing-induced residual stresses. The main geometric, material, and performance-related characteristics are summarized in Table 4.

### 3.2. FE Model Description and Analysis Setup

The structural response of the railway car body was investigated using a finite element (FE) model developed to accurately capture both global behaviour and local stress distributions. The model of the complete vehicle consisted of more than 5 million of elements. The single car body model, with reference to nodes, counted approximately 1 million nodes, providing sufficient detail for the analysis of load paths and critical regions. All structural components and subassemblies were discretized using two-dimensional, QUAD4 shell elements with first-order interpolation, which were consistently applied to both metallic and composite parts of the structure. While the same meshing strategy was adopted throughout the model, the material definition differed between conventional metallic components and laminated composite regions. In particular, the composite panels were characterized by a detailed ply-by-ply description, including material properties, thicknesses, and fibre orientations. To improve modelling efficiency and facilitate parametric and optimization studies, the laminate was defined within a dedicated local subdomain, thereby reducing redundant computations and improving control over laminate parameters. The spatial resolution of the FE model was selected to ensure an appropriate balance between numerical accuracy and computational efficiency. A reference mesh with a characteristic element length of approximately 18 mm was adopted as the baseline discretization, while finer elements were introduced locally in critical regions to better capture stress concentrations. The suitability of this choice was assessed by performing a convergence study in which the global mesh density was systematically varied over a wide range. Starting from a relatively coarse discretization with an average element size of about 25 mm, the mesh was progressively refined until a minimum size of approximately 12 mm was reached. The comparison of the resulting stress and displacement fields showed that the structural response became insensitive to further refinement at the selected resolution, indicating that the adopted mesh provides a converged and reliable representation of the system behaviour. Mechanical fasteners were modelled using an equivalent simplified representation, in which rivets were described by a combination of rigid multi-node elements (RBE2) and a one-dimensional beam element reproducing the effective cross-sectional behaviour. Major onboard equipment was represented by concentrated mass elements (CONM2) connected to the car body via RBE3 elements, allowing inertial loads to be realistically distributed over the main attachment interfaces without introducing artificial stiffness. The interface between the composite laminate and the metallic car body, corresponding to regions where adhesive bonding would be present in the physical structure, was simulated using a node-based bonded contact formulation. This approach ensured full load transfer across the interface, consistent with the methodological scope of the study. Boundary conditions were defined to reproduce an isostatic constraint configuration representative of in-service conditions. Vertical constraints were applied at the secondary suspension locations, lateral displacements were restricted at the side pads, and longitudinal motion was restrained at the rear cab buffer interfaces. According to EN 12663-1, the analysed vehicle belongs to the P-V category, which includes passenger rolling stock, focusing on light and urban rail vehicles. In addition to the static verification analyses, the finite element model was formulated to support both dynamic characterization and composite structural optimization. Starting from the full-vehicle model, each car body was extracted and analysed under free–free boundary conditions in order to identify its natural frequencies and associated mode shapes. Particular attention was devoted to the lowest vibration modes, which are known to play a key role in car body structural design and passenger comfort. The modal characterization provided essential information on the global stiffness of the structure and on the dominant deformation mechanisms, such as vertical bending, torsion, and coupled modes. The resulting modal parameters were subsequently used as reference quantities in the definition of design constraints for the laminate optimization process, ensuring that the lightweight solutions developed did not compromise the dynamic integrity of the vehicle. Concurrently, the modelling framework was configured to enable the optimization of composite laminate components. A free-size composite formulation was adopted, allowing the thickness of individual plies to vary continuously within predefined limits. This capability made it possible to evaluate mass-efficient laminate distributions while preserving consistency with both static load requirements and dynamic performance indicators derived from the modal analysis. The integration of modal characterization and free-size composite optimization within the same finite element model ensured a coherent and physically consistent basis for the development of lightweight structural solutions. Structural assessment was therefore carried out on the complete vehicle model, in accordance with the loading scenarios prescribed by the standard. Reference mass configurations were defined following EN 15663 standard, which provides a standardized framework for railway vehicle mass classification. The governing load case considered in this study corresponds to the maximum vertical loading condition: this scenario was identified as the most critical for the composite panel under investigation and corresponds to an occupancy level of six passengers per square meter, each with an assumed mass of 70 kg. Consequently, this condition was adopted as the reference case for the structural assessment and optimization of the laminated composite solution.

### 3.3. Size Optimization Formulation

The objective of the size optimization procedure is to determine the minimum thickness that satisfies both the objective function and the predefined design constraints. Following the methodology established in [50], the structural optimization problem is formulated as follows:

(1) $$Find\;\;b\in R^{n},\;z\in R^{l}$$

(2) to minimize f(b,z)

(3) $$subject\;to\;K\left(b\right)z=f$$

(4) $$g_{j}\left(b,z\right)\leq 0;\;\;j=1,\ldots ,m$$

(5) $$b_{L}\leq b\leq b_{U}$$

The optimization framework is defined by the design variable vector $b\in R^{n}\;$ and the nodal displacement vector $z\in R^{l}$, where n and l denote the number of design variables and the total degrees of freedom, respectively. The structural behaviour is governed by the linear finite element (FE) equilibrium equation $K\left(b\right)z=f$, where where K represents the global stiffness matrix and f is the vector of external loads. In this formulation, f(b) serves as the objective function, while $g_{j}$ denotes the set of m constraint functions. The design space is bounded by $b_{L}$ and $b_{U}$, representing the lower and upper limits of the design variables, respectively. The design variables are specifically mapped to the geometric properties of the composite laminates, including individual ply thicknesses. A minimum thickness threshold of 1 mm was imposed to investigate the limits of material exploitation. This constraint is supported by recent advancements in composite manufacturing, which permit the fabrication of increasingly thin laminate profiles. The optimization problem is summarized in Table 5:

## 4. Results and Discussion

### 4.1. Preliminary Static Analysis Results

**Figure 3:**
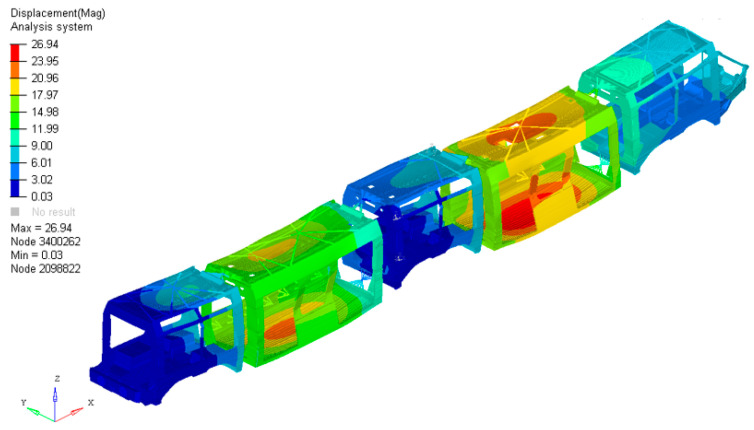
Displacement distribution of the full vehicle.

Preliminary analyses of the vehicle demonstrated mechanical behaviour consistent with expectations. As stated in previous sections, the test was conducted in accordance with European reference standards. It was performed on the full vehicle, which in this research activity is presented in its five car bodies configuration. In the application of optimization processes, it is fundamental to conduct a preliminary analysis of the system under study to establish a baseline for its original performance and identify potential areas for improvement. The displacement distribution, shown in Figure 3, highlights that the maximum values are observed in the “suspended” car body configuration. This configuration features a more elongated longitudinal geometry compared to the one analysed in this specific study, which aligns with theoretical expectations. The peak displacement values are concentrated in the floor and roof regions. Conversely, in the specific car body type investigated here, displacements are more contained due to its more compact structural design.

**Figure 4:**
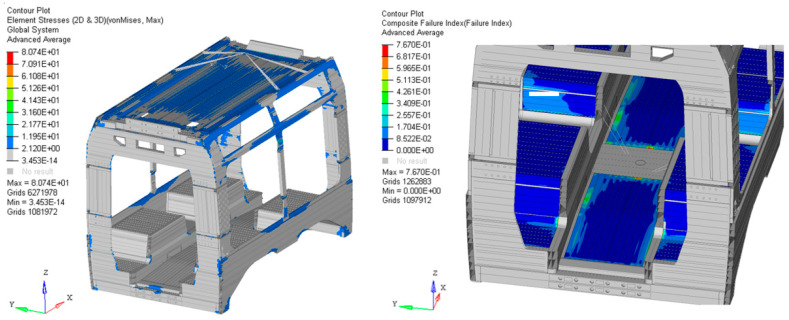
Structural response of the baseline car body: equivalent stress distribution (von Mises) for the aluminium structure (**Left**); failure index for the composite laminate components (**Right**).

Moving to the stress analysis, the car body was individually evaluated in terms of equivalent stress to assess the performance of the primary aluminium structure, and in terms of the failure index to evaluate the performance of the integrated composite laminates. Figure 4 (left) illustrates the equivalent stress distribution. The observed values are generally very low, with a peak stress of approximately 80 MPa. This value is significantly below the yield strength of the base material and most metallic alloys commonly employed in railway engineering. Regarding the composite components, shown in Figure 4 (right), the failure index, which ranges from 0 to 1, reached a maximum of 0.76. This result indicates a substantial safety margin relative to the critical threshold of 1.0, which would represent the structural failure of the laminate, opening to optimization possibilities.

**Figure 5:**
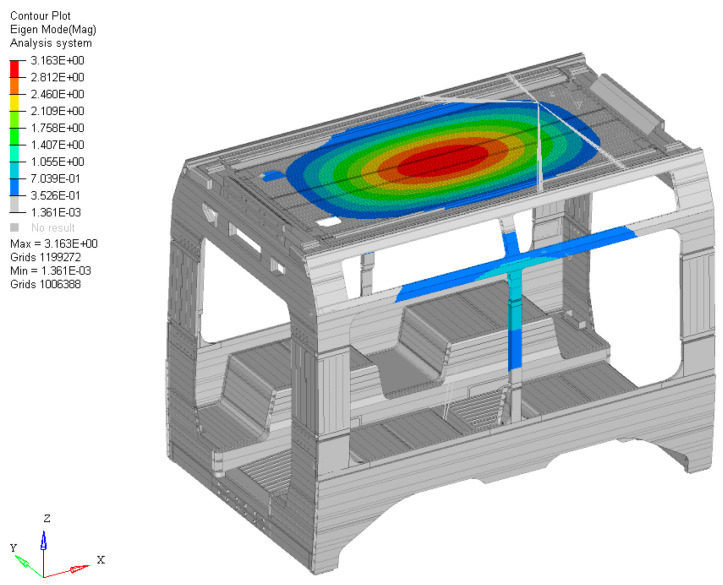
First mode shape of the baseline car body.

Finally, a free-free modal analysis was performed on the individual car body, revealing a characteristic first flexural mode localized on the roof of the structure, as illustrated in Figure 5. The first eigenvalue was identified at approximately 16 Hz. This value will serve as the primary design constraint for the first stage of the optimization process. The objective is to ensure that the optimized structure maintains, at a minimum, the dynamic performance of the original car body configuration, preventing any degradation in structural frequency response.

### 4.2. Multistage Size Optimization Results

This section presents and analyses the results obtained from the application of the proposed multistage size optimization framework on the two composite laminate configurations. The focus shifts to the effectiveness of the optimization algorithm in balancing conflicting structural requirements. Specifically, the results demonstrate how the two-stages approach enables convergence toward solutions that minimize total mass while strictly adhering to regulatory safety margins, such as maintaining target natural frequencies for vehicle passenger comfort and dynamic stability, or acceptable failure performance for the composite structure. By comparing Configuration 1 (Symmetric) and Configuration 2 (Asymmetric), it is shown that the structural optimization successfully reaches the lower bounds dictated by the dynamic constraints and the critical vertical load, providing designers with a versatile tool to explore new trade-offs between mechanical performance, lightweighting, and manufacturing costs.

#### 4.2.1. Configuration 1

For Configuration 1, the optimization process shows a drastic reduction in ply thicknesses, moving from an initial total of 3.3 mm to a final optimized state of 1 mm. First, the process begins with Iteration 0, which immediately shows a light reduction from 3.3 mm to 2.820 mm. The detailed evolution of these thicknesses across the optimization iterations is summarized in Table 6. This suggests the initial design was significantly conservative. Comparing the original thickness of Ply 1, 0.250 mm, with the final Iteration 2 value, 0.076 mm, it is possible to observe a thickness reduction of approximately 69.6%. This trend is consistent across all layers; for instance, the glass fibre plies dropped from 0.350 mm to 0.106 mm, with a reduction of 69.7%. As detailed in Table 7, during the static optimization stages, thickness evolves across Iterations 1 and 2, but eventually is bounded below by a total thickness of 1 mm. This value was predefined by the model-optimization stage as the minimum threshold to maintain the car body’s natural frequency. The fact that the static optimizer stops exactly at this dynamic limit proves that for this configuration, the stiffness required for vibration control is more demanding than the strength required for static loads.

#### 4.2.2. Configuration 2

In Configuration 2, characterized by a lower carbon fibre content to reduce manufacturing costs, the optimization demonstrates a similar but distinct patch. Just as in the Configuration 1, Iteration 0 provides an immediate thinning to 2.560 mm (as 14.6% reduction), this indicates that even a cost-optimized baseline has immediate room for improvement. Starting from a total of 3 mm, the final thickness reaches the same 1 mm limit, representing a total reduction of 66.7%. Notably, Ply 10 (carbon fibre) was reduced from 0.050 mm to 0.017 mm, corresponding to a reduction of approximately 66%. Despite being lower, as it is possible to see for Configuration 1, in Configuration 2, the optimization allows the material to reach its physical limits and the multistage process ensures that safety is never compromised, while the thicknesses are pushed down to the 1 mm dynamic boundary. The results of this analysis are presented in Table 8 and Table 9.

#### 4.2.3. Final Comparative Analysis and Discussion

**Figure 6:**
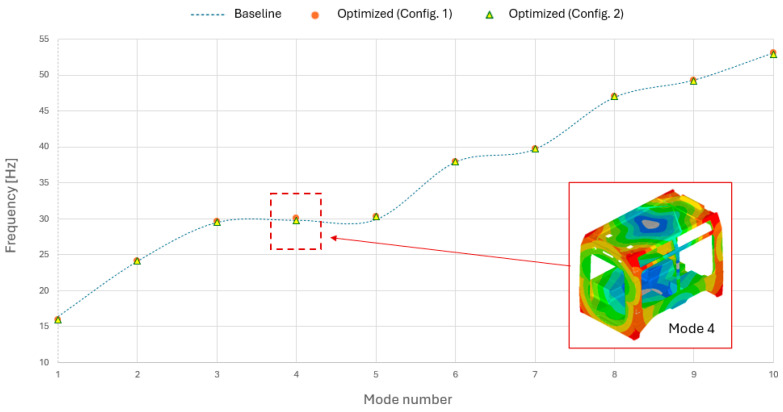
Comparative distribution of the first ten natural frequencies: baseline car body vs. optimized symmetric (Configuration 1) and asymmetric (Configuration 2) configurations.

The comparison between the optimization outcomes of Configuration 1 and Configuration 2 provides significant insights into the efficiency of the proposed multistage framework and the structural behaviour of the hybrid car body. A comprehensive re-analysis was performed on both tested configurations in strict accordance with the reference standard. Regarding global displacements, no significant deviations were observed compared to the baseline configuration. Similarly, the primary aluminium structure exhibited nearly identical stress levels following the optimization of the two composite laminates. To maintain a concise discussion and given the absence of critical variations, these specific results will not be further detailed. This outcome confirms, from both a static and dynamic perspective, that the influence of the integrated composite laminates is a local effect, without negatively impacting the global structural performance. This outcome is clearly illustrated in Figure 6, which provides a comparative modal analysis between the baseline configuration and the two optimized designs (Configuration 1 and Configuration 2). The results demonstrate that the eigenfrequency variations for the first ten vibration modes are practically negligible. This is primarily because the majority of these modes are localized on the roof structure, thus remaining unaffected by the modifications. However, the most significant variation is observed in Mode 4. In this instance, the mode shape exhibits a global character, involving the regions where the composite laminates are integrated. This interaction is clearly visualized in the figure, highlighting how structural optimization specifically influences modes that engage the reinforced zones. Both configurations fully satisfied the frequency constraints, ensuring a fundamental frequency (first eigenvalue) of approximately 16 Hz. As previously detailed, both designs achieved a thickness reduction of roughly 66% for the composite laminates. Notably, this value reached the lower bound of the allowable range defined by the design variables, indicating that the optimization successfully converged toward the most mass-efficient solution possible within the established design space.

**Figure 7:**
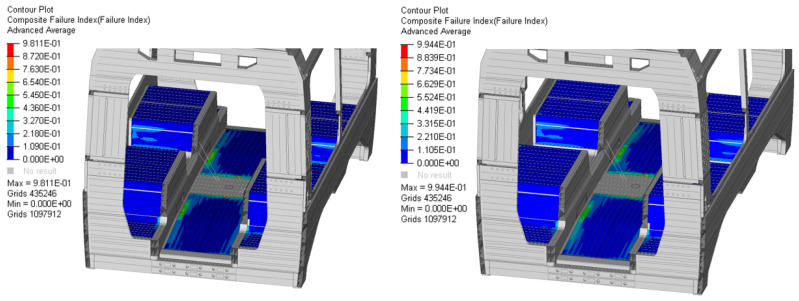
Structural performance of the optimized car body configurations: comparison of failure indices (FIs) for Configuration 1 and Configuration 2.

Achieving a nearly negligible shift in natural frequencies represents a significant milestone in the design process. This stability allows for a more aggressive pursuit of lightweight design and the optimization of mechanical performance without compromising the structural dynamic behaviour. Consequently, the mass reduction and structural tailoring of the composite laminates do not adversely affect the in-service dynamic performance of the car body shell. This ensures that the global vibration response and stability remain consistent with the original design requirements, despite the introduction of advanced material solutions. Focusing on the mechanical performance of the two configurations, Figure 7 illustrates that the most critical stress distributions are located on the floor section. These results are consistent with the findings from the baseline analysis. The optimization process enabled a significant reduction in laminate thickness while remaining strictly compliant with the failure index (FI) constraints. Compared to the preliminary tests, a thickness reduction of approximately 66% was achieved. This resulted in a controlled increase in the failure index by 22.5% for Configuration 1 and 23.3% for Configuration 2, showing a better utilization coefficient. From a material behaviour perspective, this increase in the FI represents a transition toward a more efficient exploitation of the laminate’s load-bearing capacity. The failure mechanism is primarily dominated by in-plane stresses, with the optimized stacking sequences ensuring that the fibre-dominated strength is maximized before reaching the matrix degradation threshold. By reducing the over-dimensioned baseline thickness, the framework redistributes the internal strain energy more uniformly across the plies, avoiding localized stress concentrations that could lead to early delamination or ply cracking. The marginal difference between the two stems from the specific stacking sequences and laminate architectures investigated; nonetheless, both configurations demonstrated good mechanical performance and highly effective lightweight design characteristics.

## 5. Conclusions

This study presented a robust multistage optimization framework for the integration of composite laminates into a low-floor light rail vehicle car body, developed in strict accordance with reference European standards. The research addressed the inherent structural challenges of low-floor architectures, characterized by complex load paths and redistributed equipment masses, by leveraging the high specific stiffness of advanced composite materials. To validate the flexibility of the proposed methodology, two distinct composite configurations were investigated: a symmetric stacking sequence to eliminate extension–bending coupling, and an asymmetric layup to evaluate the algorithm’s performance under more complex constitutive behaviours and coupling effects. The main results of the proposed multistage optimization can be summarized as follows:

- The framework successfully integrated both performance requirements into a single, cohesive methodology. Regarding dynamic stability, the car body integrity remained uncompromised, despite the reduction in mass.
- Both configurations maintained a fundamental natural frequency of approximately 16 Hz, ensuring the first vertical bending mode remains well above the critical thresholds for passenger discomfort (typically <10 Hz). A very high correlation was observed across the first ten vibration modes, confirming that the overall dynamic behaviour remains consistent and predictable post-optimization.
- The approach enabled a thickness reduction of approximately 66% for both laminate configurations. While this led to an increase in the failure index (FI) of 22.5% for Configuration 1 and 23.3% for Configuration 2, the results remained fully compliant with regulatory safety margins, maximizing material utilization.

In conclusion, the adopted multistage optimization approach demonstrates its effectiveness in converging toward a solution that simultaneously accounts for both dynamic and static requirements. Given the specific placement of the laminates, it was observed that the optimal solution for the static optimization aligns exactly with the lower bound of the allowable range imposed by the dynamic constraints. This result is particularly significant, as it suggests that designers can explore solutions with slightly lower mechanical targets to achieve an even more favourable trade-off between manufacturing costs, weight reduction, and overall structural performance.

## Figures and Tables

**Table 1:** Mechanical properties of plies—Configuration 1.

Ply	Material	Thickness [mm]	Density [kg/m^3^]	E_1_ [GPa]	E_2_ [GPa]	ν_12_ [GPa]	G_12_ [GPa]	G_13_ [GPa]	G_23_ [GPa]
1	Carbon fibre/Epoxy	0.250	1590	135	8.0	0.30	5.0	5.0	3.0
2	Glass fibre/Epoxy	0.350	2010	40	10.0	0.25	4.5	4.5	3.5
3	Glass fibre/Epoxy	0.350	2010	40	10.0	0.25	4.5	4.5	3.5
4	Glass fibre/Epoxy	0.350	2010	40	10.0	0.25	4.5	4.5	3.5
5	Carbon fibre/Epoxy	0.350	1590	135	8.0	0.30	5.0	5.0	3.0
6	Carbon fibre/Epoxy	0.350	1590	135	8.0	0.30	5.0	5.0	3.0
7	Glass fibre/Epoxy	0.350	2010	40	10.0	0.25	4.5	4.5	3.5
8	Glass fibre/Epoxy	0.350	2010	40	10.0	0.25	4.5	4.5	3.5
9	Glass fibre/Epoxy	0.350	2010	40	10.0	0.25	4.5	4.5	3.5
10	Carbon fibre/Epoxy	0.250	1590	135	8.0	0.30	5.0	5.0	3.0

**Table 2:** Mechanical properties of plies—Configuration 2.

Ply	Material	Thickness [mm]	Density [kg/m^3^]	E_1_ [GPa]	E_2_ [GPa]	ν_12_ [GPa]	G_12_ [GPa]	G_13_ [GPa]	G_23_ [GPa]
1	Carbon fibre/Epoxy	0.200	1590	135	8.0	0.30	5.0	5.0	3.0
2	Glass fibre/Epoxy	0.400	2010	40	10.0	0.25	4.5	4.5	3.5
3	Glass fibre/Epoxy	0.350	2010	40	10.0	0.25	4.5	4.5	3.5
4	Glass fibre/Epoxy	0.350	2010	40	10.0	0.25	4.5	4.5	3.5
5	Glass fibre/Epoxy	0.400	1590	135	8.0	0.30	5.0	5.0	3.0
6	Glass fibre/Epoxy	0.400	1590	135	8.0	0.30	5.0	5.0	3.0
7	Glass fibre/Epoxy	0.350	2010	40	10.0	0.25	4.5	4.5	3.5
8	Glass fibre/Epoxy	0.350	2010	40	10.0	0.25	4.5	4.5	3.5
9	Glass fibre/Epoxy	0.150	2010	40	10.0	0.25	4.5	4.5	3.5
10	Carbon fibre/Epoxy	0.050	1590	135	8.0	0.30	5.0	5.0	3.0

**Table 3:** Failure parameters values of composite materials.

Material	Xt [MPa]	Xc [MPa]	Yt [MPa]	Yc [MPa]	S [MPa]	τ_il [MPa]
Carbon fibre/Epoxy	1500	800	60	160	80	60
Glass fibre/Epoxy	400	250	80	150	50	40

**Table 4:** Geometrical and material comparison between laminate configurations.

Feature	Configuration 1 [Symmetric]	Configuration 2 [Asymmetric]
Total thickness [mm]	3.3	3
Carbon content	36.36%	8.33%
Glass content	63.64%	91.67%

**Table 5:** Optimization settings.

Objective Function	Minimization of the total car body mass
Class of optimization	Free-size optimization
Design Variables	Thicknesses of the composite laminates
Design Constraint (stage 1)	1st freq (optimized car body) > 1st freq (original car body)
Design Constraint (stage 2)	Failure index of the composite laminates < 1

**Table 6:** Ply-by-ply thickness evolution during optimization: Stage 1, Configuration 1.

Ply	Material	Original Thickness [mm]	Thickness to Iteration 0 [mm]	Thickness to Iteration 1 [mm]	Thickness to Iteration 2 [mm]
1	Carbon fibre	0.250	0.225	0.150	0.076
2	Glass fibre	0.350	0.315	0.210	0.106
3	Glass fibre	0.350	0.315	0.210	0.106
4	Glass fibre	0.350	0.315	0.210	0.106
5	Carbon fibre	0.350	0.315	0.210	0.106
6	Carbon fibre	0.350	0.315	0.210	0.106
7	Glass fibre	0.350	0.315	0.210	0.106
8	Glass fibre	0.350	0.315	0.210	0.106
9	Glass fibre	0.350	0.315	0.210	0.106
10	Carbon fibre	0.250	0.225	0.150	0.076
Total thickness		3.300	2.970	1.980	1.000

**Table 7:** Ply-by-ply thickness evolution during optimization: Stage 2, Configuration 1.

Ply	Material	Original Thickness [mm]	Thickness to Iteration 0 [mm]	Thickness to Iteration 1 [mm]	Thickness to Iteration 2 [mm]
1	Carbon fibre	0.250	0.210	0.125	0.076
2	Glass fibre	0.350	0.300	0.190	0.106
3	Glass fibre	0.350	0.300	0.190	0.106
4	Glass fibre	0.350	0.300	0.190	0.106
5	Carbon fibre	0.350	0.300	0.190	0.106
6	Carbon fibre	0.350	0.300	0.190	0.106
7	Glass fibre	0.350	0.300	0.190	0.106
8	Glass fibre	0.350	0.300	0.190	0.106
9	Glass fibre	0.350	0.300	0.190	0.106
10	Carbon fibre	0.250	0.210	0.125	0.076
Total thickness		3.300	2.820	1.770	1.000

**Table 8:** Ply-by-ply thickness evolution during optimization: Stage 1, Configuration 2.

Ply	Material	Original Thickness [mm]	Thickness to Iteration 0 [mm]	Thickness to Iteration 1 [mm]	Thickness to Iteration 2 [mm]
1	Carbon fibre	0.200	0.180	0.120	0.066
2	Glass fibre	0.400	0.360	0.240	0.133
3	Glass fibre	0.350	0.315	0.210	0.117
4	Glass fibre	0.350	0.315	0.210	0.117
5	Carbon fibre	0.400	0.360	0.240	0.133
6	Carbon fibre	0.400	0.360	0.240	0.133
7	Glass fibre	0.350	0.315	0.210	0.117
8	Glass fibre	0.350	0.315	0.210	0.117
9	Glass fibre	0.150	0.135	0.090	0.050
10	Carbon fibre	0.050	0.045	0.030	0.017
Total thickness		3.000	2.700	1.800	1.000

**Table 9:** Ply-by-ply thickness evolution during optimization: Stage 2, Configuration 2.

Ply	Material	Original Thickness [mm]	Thickness to Iteration 0 [mm]	Thickness to Iteration 1 [mm]	Thickness to Iteration 2 [mm]
1	Carbon fibre	0.200	0.170	0.110	0.066
2	Glass fibre	0.400	0.340	0.220	0.133
3	Glass fibre	0.350	0.300	0.190	0.117
4	Glass fibre	0.350	0.300	0.190	0.117
5	Carbon fibre	0.400	0.340	0.220	0.133
6	Carbon fibre	0.400	0.340	0.220	0.133
7	Glass fibre	0.350	0.300	0.190	0.117
8	Glass fibre	0.350	0.300	0.190	0.117
9	Glass fibre	0.150	0.130	0.080	0.050
10	Carbon fibre	0.050	0.040	0.025	0.017
Total thickness		3.000	2.560	1.635	1.000

## Data Availability

The original contributions presented in this study are included in the article. Further inquiries can be directed to the corresponding author.
